# B cell subsets phenotype in autoimmunity with immunodeficiency: analysis of a cohort of patients with APECED syndrome

**DOI:** 10.1186/1546-0096-9-S1-P285

**Published:** 2011-09-14

**Authors:** A Magnani, A Meloni, M Gattorno, A Martini, E Traggiai

**Affiliations:** 1 Laboratory of Immunology of Rheumatic Diseases,U.O. Pediatria II, G. Gaslini Institute and Department of Pediatrics, University of Genoa, Italy; 2 Pediatric Clinic II, Ospedale Microcitemico and Department of Biomedical and Biotechnological Science, University of Cagliari, Italy

## Background

Autoimmune polyendocrinopathy candidiasis ectodermal dystrophy (APECED) is a rare autosomal recessive syndrome due to mutations in *AIRE*, characterized by autoimmune endocrinopathies and mucocutaneous candidiasis. It is accompanied by serum auto-antibodies whose generation has been mainly related to autoreactive T cells escape from tolerance mechanisms. Recent data suggest a T cell independent mechanism implicated in altered peripheral B cell selection and its possible contribution to the pathogenesis of autoimmune disease. Despite these observations, B cell subsets phenotype in these patients is still poorly characterized.

## Aim

To perform a detailed analysis of B and T cell subsets in a cohort of APECED patients in order to better understand whether an intrinsic alteration in B cell compartment is present. We also evaluated B lymphocytes related cytokines (BAFF, IL-21) in patients’ sera compared to age matched control.

## Methods

Flow cytometric analysis of B cell subsets was performed in 12 patients with APECED coming from Sardinia, Italy, compared to age-matched healthy donors. The following B cell subsets were analysed: transitional, naïve, IgM and switched memory and plasmacells; T cell subsets: Treg, CD4 and CD8 T cell subsets (naïve, central memory, effector memory) ELISA assay was performed for cytokines determination in sera.

## Results

Analysis show an altered distribution of the immature peripheral B cells compartment in APECED patients and an increased level of BAFF in patients’ sera. The T cell compartment is skewed towards effector memory T cells.

## Conclusions

Increasing the knowledge on B cells in APECED patients improves the comprehension of autoimmunity pathogenesis in immunodeficiency and may allow the exploration of the possible clinical efficacy of B cell targeted therapy.

